# Retained Epidural Catheter Fragment Requiring Surgical Removal in a Patient With Multiple Comorbidities: A Case Report

**DOI:** 10.7759/cureus.105932

**Published:** 2026-03-26

**Authors:** Jose Gerardo Lopez Saenz, Chuang Lin Wang Kong, Alexa Salazar, Alonso Hidalgo, Emmanuelle Vargas

**Affiliations:** 1 Anesthesia, Hospital México, San José, CRI; 2 Medicine, University of Costa Rica, San José, CRI; 3 Neurosurgery, Hospital México, San José, CRI

**Keywords:** broken epidural catheter, epidural analgesia, epidural catheter complication, equipment failure, foreign bodies

## Abstract

Epidural analgesia remains an important technique in many clinical settings; although generally considered safe, it is not free of complications. We present the case of a 69-year-old woman with poorly controlled diabetes mellitus and hypertension who, during an epidural block for a left open hepatectomy, experienced the fracture and retention of a 10-cm epidural catheter fragment within the spinal canal at the T12-L1 level. Despite the patient being asymptomatic, surgical removal was required due to the fragment's location and length and the potential risk of long-term systemic complications associated with her comorbidities. Among the main causes of catheter retention, excessive traction during removal is one of the most frequently reported mechanisms. The management of a retained epidural catheter fragment depends on multiple variables, including fragment size, anatomical location, symptomatology, and patient comorbidities. While conservative management may be appropriate for selected asymptomatic patients with small fragments located outside the spinal canal, surgical extraction is recommended when the fragment is large, located within the spinal canal, and associated with an increased risk of infection or systemic complications or when the patient develops neurological symptoms.

## Introduction

Epidural is a widely used technique for labor analgesia, orthopedic procedures, and major abdominal surgery due to its benefits in providing intraoperative anesthesia and reducing systemic postoperative complications [1,2]. In this technique, a catheter made of polyethylene is inserted through the skin into the epidural space, and pain-relieving medications, such as local anesthetics or opioids, are administered as boluses or via continuous infusion [3].

However, this procedure is not free of complications, which include catheter migration, epidural hematomas or abscesses, and neurological injury [4]. Although extremely rare, complications such as the fracture and retention of an epidural catheter may also occur, with an estimated incidence ranging from 0.002% to 0.07% [4,5]. The management of retained fragments remains controversial and depends on patient-specific factors, including age, symptoms, fragment location, and the risk of infection [1,2].

We present a case of a fractured polyethylene epidural catheter in a patient with multiple metabolic comorbidities and discuss the mechanisms underlying this complication, the role of catheter material, and the clinical decision-making process regarding its surgical removal.

## Case presentation

A 69-year-old female patient (52 kg), an active smoker, with a history of poorly controlled type 2 diabetes mellitus, hypertension, dyslipidemia, osteoporosis, hypothyroidism, and left breast cancer, was admitted for a left hepatectomy due to suspected metastasis associated with her previous malignancy. Preoperative laboratory tests showed a normal complete blood count and coagulation profile; however, her diabetes mellitus was decompensated, with a glycated hemoglobin (HbA1c) level of 8.5%.

Neuraxial analgesia via an epidural block and catheter placement was selected for postoperative pain management. Under strict aseptic conditions and with the patient in the seated position, the epidural space was approached at the T12-L1 interspace using an 18-gauge Tuohy needle (Perifix® 421 set, B. Braun, Melsungen, Germany). After two initial attempts, the epidural space was successfully identified using the loss-of-resistance to air technique at a depth of 5 cm from the skin. 

**Figure 1:**
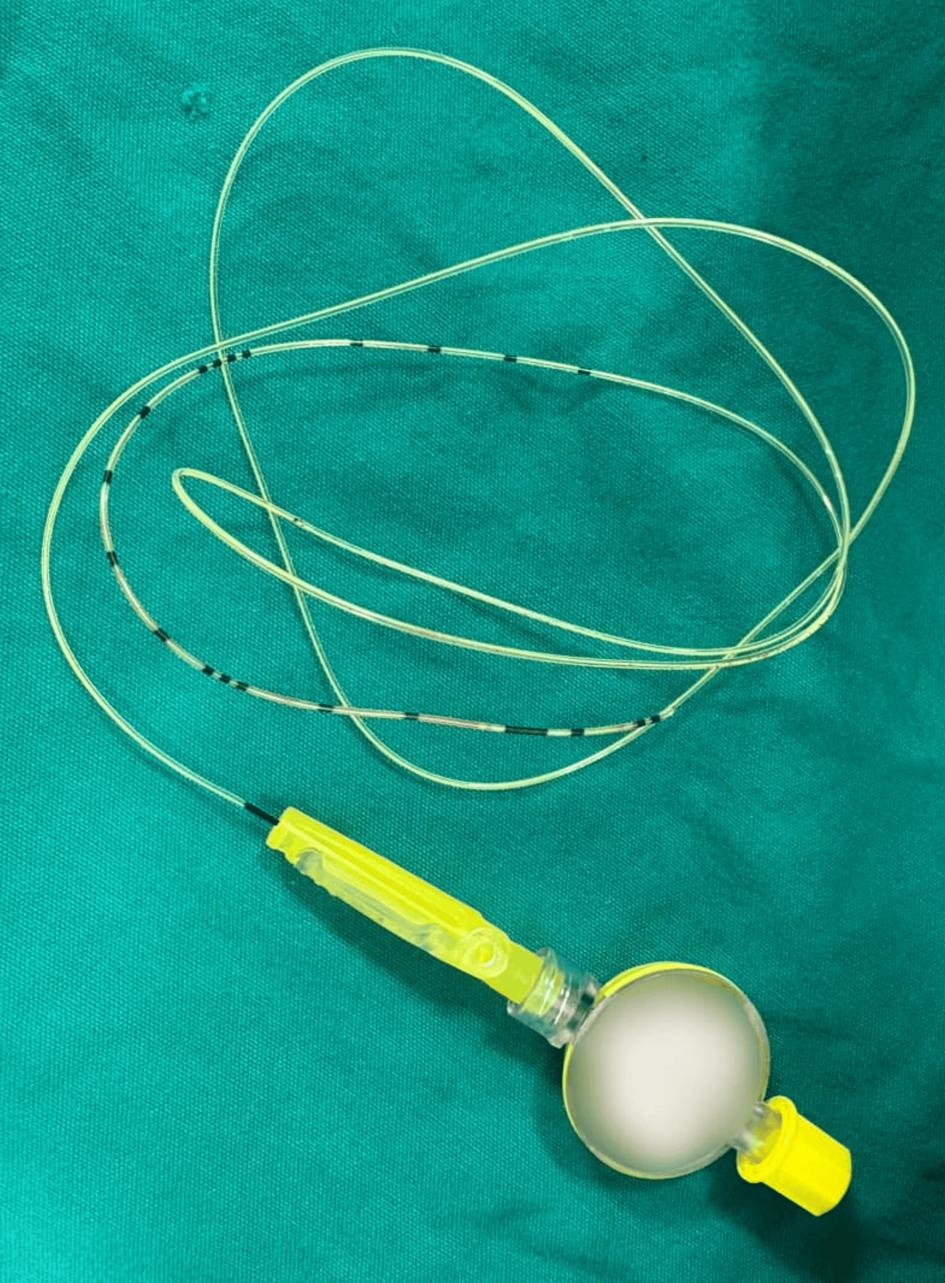
Fractured epidural catheter.

The catheter was advanced into the epidural space without further abnormal resistance. Aspiration for blood and cerebrospinal fluid (CSF) was negative. The Tuohy needle was then withdrawn without resistance or complications. However, as the catheter was being repositioned to the clinical objective of 7-8 cm, the epidural catheter ruptured despite the application of only gentle traction. This resulted in an approximately 10-cm fragment being retained within the patient (Figure 1).

**Figure 2:**
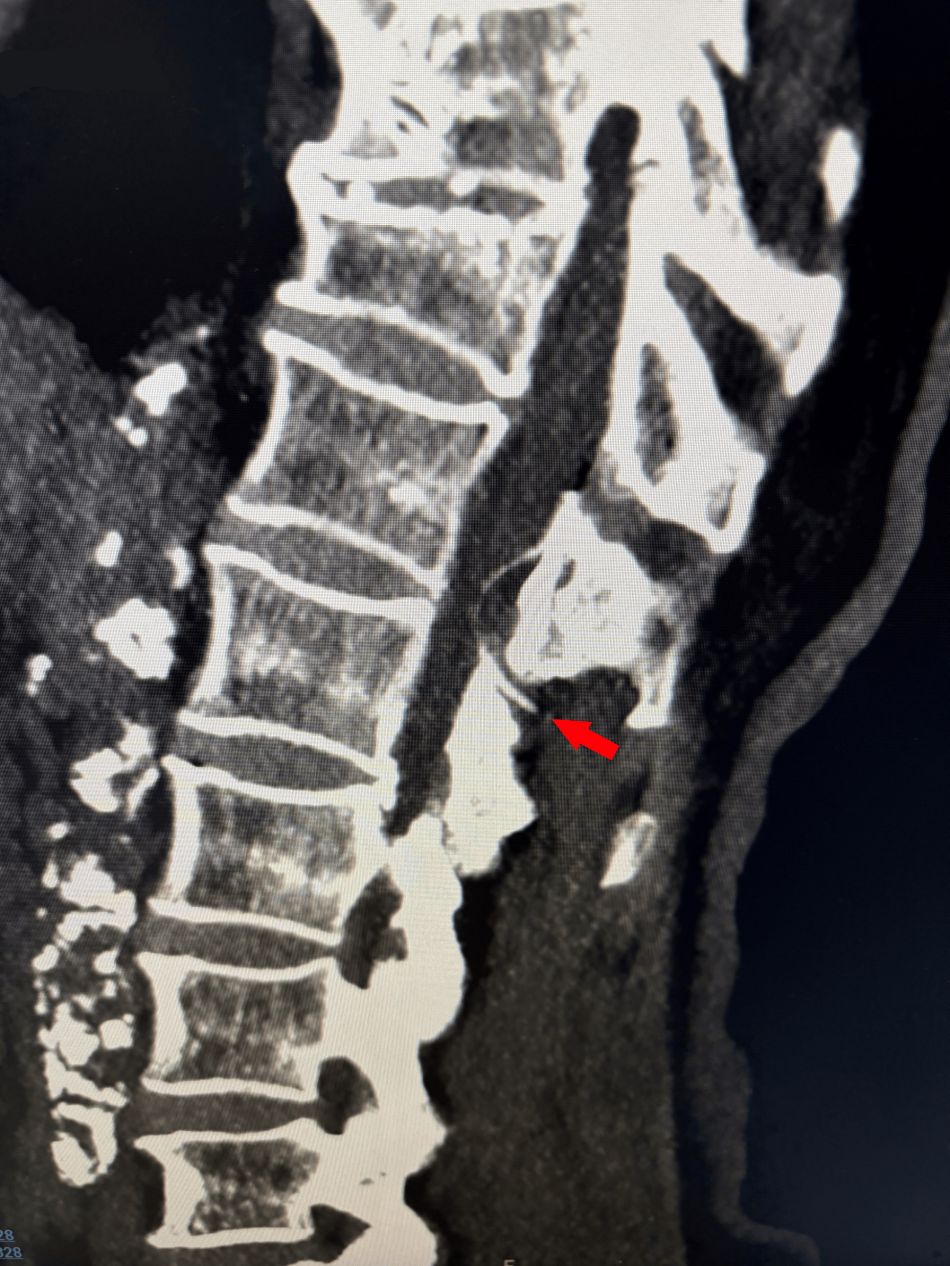
Spinal CT scan showing a fragment of the epidural catheter at the T12-L1 level. CT: computed tomography

The procedure was immediately aborted, and a computed tomography (CT) scan confirmed the presence of a catheter fragment at the T12-L1 level within the spinal canal (Figure 2). The patient remained asymptomatic, reporting no pain at the insertion site, no lower limb paresthesia, and no other neurological deficits. Physical examination revealed no signs of local infection or active bleeding.

A multidisciplinary discussion was held between the anesthesiology and neurosurgery teams. Given the patient's high risk of infection due to poorly controlled metabolic comorbidities, the decision was made to proceed with surgical removal.

**Figure 3:**
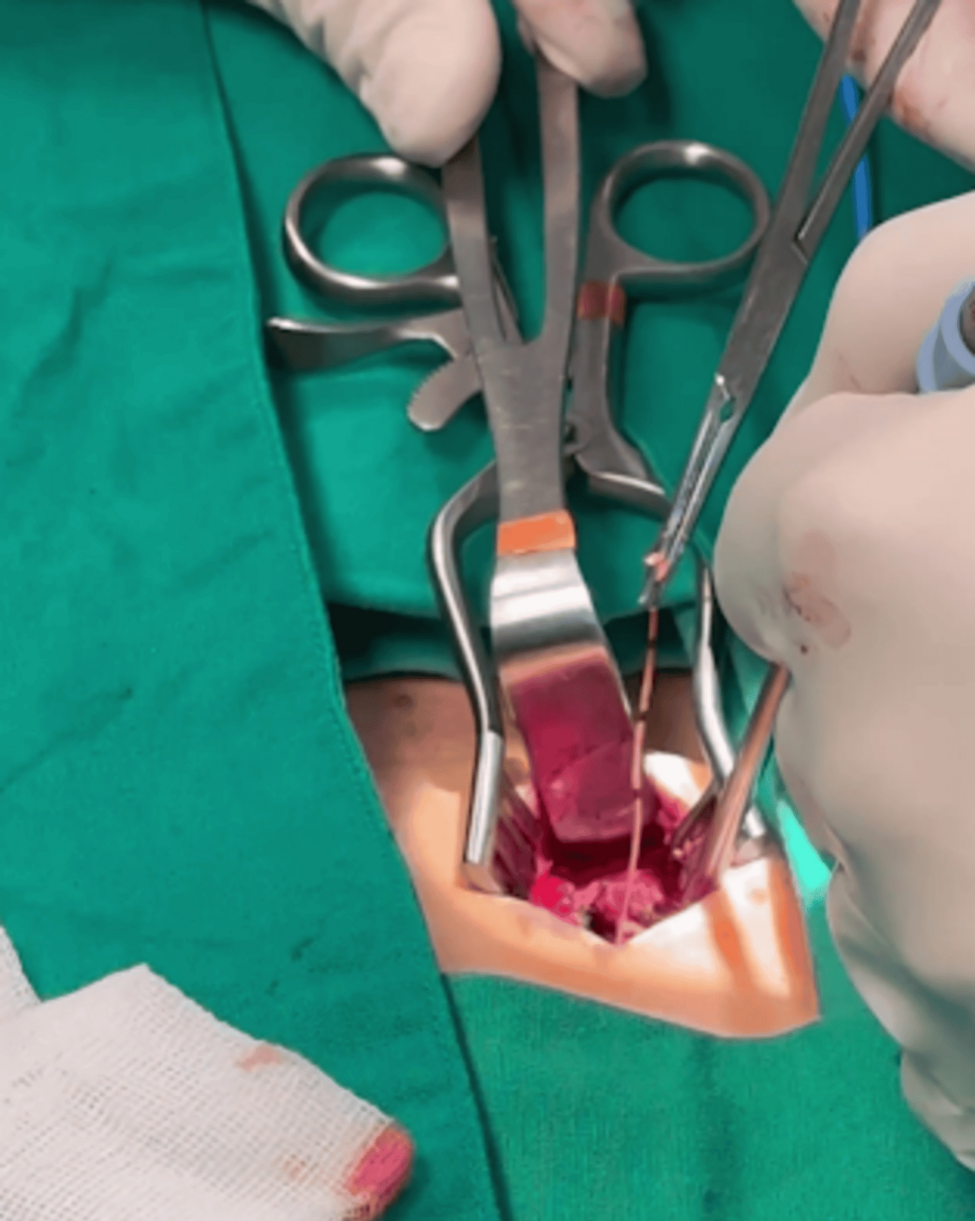
Intraoperative view of surgical exploration with the extraction of the retained catheter fragment.

**Figure 4:**
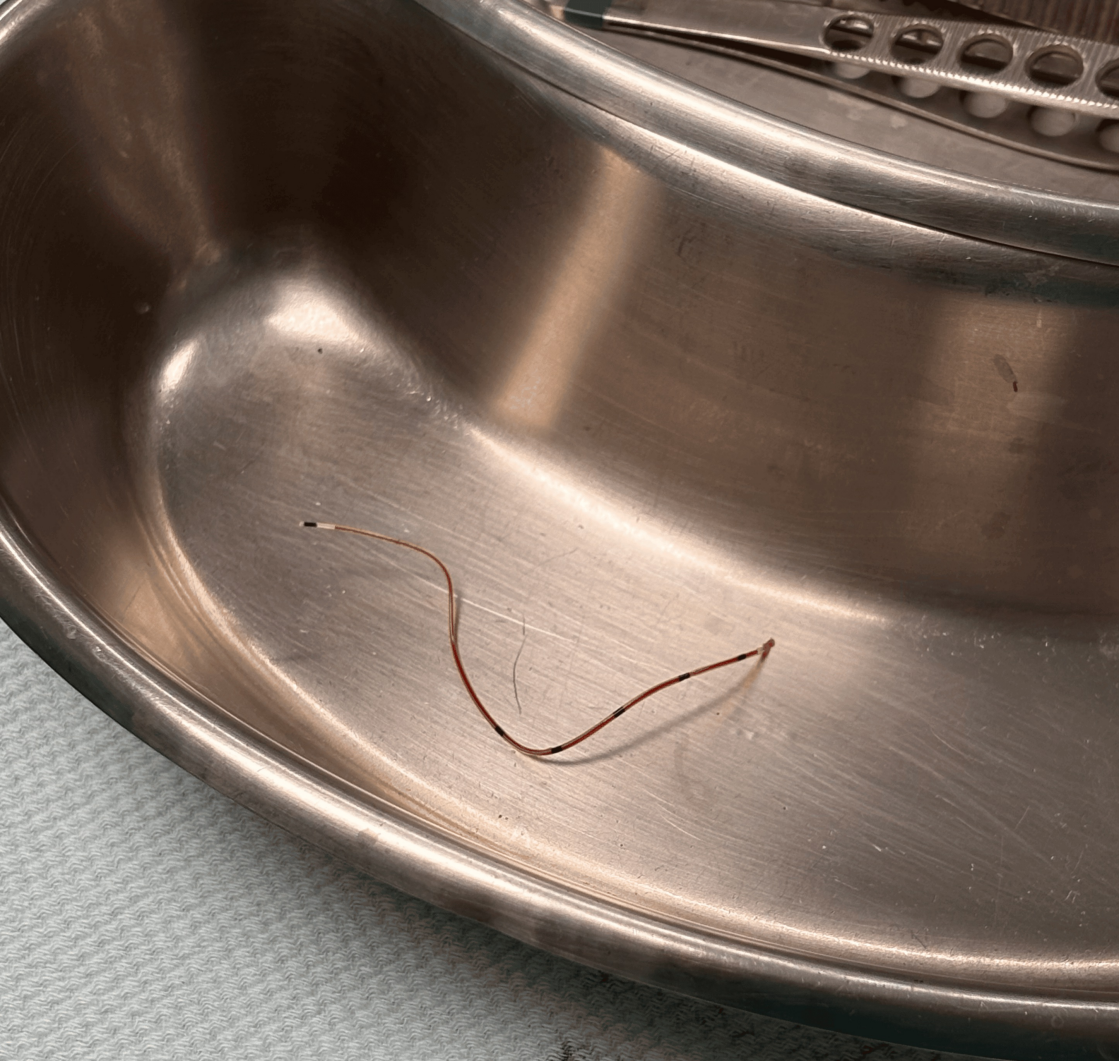
Extracted fragment of the epidural catheter.

The patient was taken to the operating room the same day, where the neurosurgery team performed paraspinal exploration at the affected vertebral level. The retained spinal catheter fragment was successfully removed without intraoperative or immediate postoperative complications (Figure 3 and Figure 4).

## Discussion

Epidural analgesia through catheter placement in the epidural space remains a fundamental technique in many clinical scenarios. In hepatectomies, such as the one described in the present case, thoracic epidural anesthesia has proven to be an effective technique for both perioperative and postoperative pain management, with an impact on the regulation of the surgical stress response, reduction of systemic complications, and decreased opioid consumption, among other benefits. However, as an invasive technique, it requires a high level of technical skill, as it has been associated with complications such as epidural hematoma, epidural abscess, and catheter retention due to fracture [6,7]. The breakage of an epidural catheter, first described in 1957, is a rare yet clinically significant complication [1,2].

Epidural catheter fracture can occur through multiple mechanisms associated with insertion-related trauma, extraction-related trauma, or anatomical factors. During insertion, several mechanisms may contribute to catheter breakage, including excessive force used to advance the catheter, insertion of an excessive catheter length, advancement of the needle over the catheter, multiple insertion attempts, and withdrawal of the catheter through the Tuohy needle. At the time of withdrawal, excessive traction force may fracture the catheter; this mechanism has been the most frequently documented in reported cases of this complication and is the cause considered in the present case [1].

Once in the epidural space, the catheter interacts with surrounding anatomical structures, which may contribute to its breakage, including adhesions, coiling, and knotting around fascia, ligaments, bony structures, or nerves, all of which may increase mechanical stress during extraction. Additional factors that may be involved include manufacturing defects, operator-related errors, and material-related mechanical properties. Regarding catheter composition, modern epidural catheters are typically manufactured from materials such as nylon, polyurethane, and polyethylene. The mechanical properties of these materials, including tensile strength, flexibility, and resistance to shear forces, have been shown to influence susceptibility to complications such as the one presented in this case. Polyurethane catheters have been reported to exhibit greater tensile strength and flexibility compared to other materials, which may reduce the risk of mechanical failure. Variations in these properties among other catheter materials, such as polyethylene, contribute to lower resistance and a higher susceptibility to kinking, shearing, and fracture. In the present case, the catheter used was made of polyethylene, which, as previously discussed, does not demonstrate the highest mechanical performance among available materials; therefore, it may have been a contributing factor to the fracture observed in this patient [1,2,8,9].

For management, initial documentation of the retained fragment is essential. Magnetic resonance imaging (MRI) is the primary imaging modality recommended, as it provides the highest discriminative performance, with a detection rate of 71.4% [8]. CT scan detects approximately 56% of fragmented catheters, and its detectability is influenced by catheter length and material composition [8]. CT scans are recommended when MRI findings are inconclusive or when MRI is not readily available, as occurred in the present case. There have also been reports of fragment identification using plain radiography; however, visibility is highly dependent on catheter material [1,2,8]. In the case presented, the catheter fragment was documented using a CT scan, as it was the only imaging modality available at the time.

According to Gompels et al., surgical intervention is not required when imaging confirms that the fragment lies completely outside the spinal canal and no portion breaches the epidermis that could serve as a portal of infection [10]. Tan et al. further propose that conservative management may be considered in an asymptomatic patient and a small retained fragment (<5 cm) [2]. In such cases, close follow-up is mandatory, and patients must be counseled regarding warning symptoms. Nonetheless, up to 39% of patients initially managed conservatively may ultimately require delayed surgical intervention due to long-term complications, including spinal stenosis from scar formation, CSF leakage, catheter migration, nerve entrapment with radicular pain, subdural hematoma, or abscess formation [1,11,12]. The present case involves a 69-year-old woman with multiple comorbidities and a 10-cm retained catheter fragment located within the spinal canal. Although the absence of symptoms and infection might support conservative management, the intraspinal location and substantial fragment length weighed against this approach.

On the other hand, indications favoring surgical management include intraspinal location of the fragment, exposure of the fragment creating a potential fistulous tract, retained length greater than 5 cm, intrathecal migration, or the presence of symptoms such as low back pain, radicular pain, paresthesia, urinary incontinence, paralysis, headache, local swelling or erythema, or other signs of infection [2,10]. Corr et al. further emphasize that patients at high risk of complications, particularly infection, should be considered for early surgical removal. When indicated, removal within days is recommended to minimize dural adhesions, granuloma formation, and reactive fibrosis, which may increase procedural complexity [1,2,10].

In this case, both the length and location of the fragment supported a surgical approach. In addition, her metabolic comorbidities, including poorly controlled diabetes mellitus, dyslipidemia, and hypertension, increase the risk of future infectious complications [10,13]. Suspected active malignancy and ongoing tobacco use further increase the likelihood of delayed systemic complications. Collectively, these factors supported early surgical removal [1,2,10]. Patient preference should also be incorporated into decision-making, as retained epidural catheter fragments may generate significant psychological distress [1,2].

Given that this represents a clinically relevant complication, preventive strategies are essential. First, the procedure should be performed by a skilled operator or under the supervision of an experienced clinician. During insertion, only 4-5 cm of the catheter should be advanced into the epidural space to prevent coiling, and if an additional attempt becomes necessary, the catheter should always be withdrawn together with the Tuohy needle. During removal, if resistance is encountered, placing the patient in the same position as during insertion or in a flexed lateral decubitus position has been shown to be more effective. If difficulty persists, a 15-30-minute pause is recommended to promote tissue relaxation before attempting removal again [2,8].

Despite advances across multiple fields of medicine, the management of retained epidural catheters remains controversial for several reasons. First, as this is a rare complication of the technique, most of the available evidence comes from small retrospective case series or isolated case reports [2,5]. The lack of clinical trials evaluating the prevalence of this complication across different catheter brands, insertion techniques, and patient profiles, together with the absence of long-term follow-up, limits the ability to establish robust, evidence-based recommendations to guide the choice between conservative and surgical management [1,5,7].

Additionally, published reports are likely affected by reporting bias, as successful surgical cases and severe complications are more frequently described, whereas asymptomatic cases managed conservatively may be underreported [2,8]. Consequently, there is no clear consensus regarding follow-up strategies for asymptomatic patients. Unresolved issues include the optimal frequency of follow-up visits, whether routine imaging is necessary or whether clinical monitoring alone is sufficient, and the appropriate duration of surveillance, given that delayed subdural hematoma has been reported even 18 years after catheter fragment retention [1,8]. Further research is needed to better characterize the underlying biological response to catheter polymers, particularly the local immune and fibrotic response, which may stimulate the development of improved biomaterials with lower long-term complication risk.

## Conclusions

Fracture and retention of an epidural catheter is a rare but clinically significant complication requiring individualized management. Decision-making should consider fragment size, anatomical location, symptomatology, and patient-specific risk factors, particularly the risks of infection and neurological compromise.

Even in asymptomatic patients, surgical removal may be the most appropriate strategy when the fragment is located within the spinal canal or exceeds 5 cm in length or when comorbidities increase the risk of delayed complications. A multidisciplinary approach is essential to optimize outcomes. Further research is warranted to establish standardized management and follow-up strategies.
